# PCR detection of *Plasmodium falciparum *in human urine and saliva samples

**DOI:** 10.1186/1475-2875-5-103

**Published:** 2006-11-08

**Authors:** Sungano Mharakurwa, Christopher Simoloka, Philip E Thuma, Clive J Shiff, David J Sullivan

**Affiliations:** 1 The Malaria Institute at Macha, P.O. Box 630166, Choma, Zambia; 2 Department of Molecular Microbiology & Immunology, Johns Hopkins Bloomberg School of Public Health, 615 N. Wolfe Street, Baltimore MD 21205, USA

## Abstract

**Background:**

Current detection or screening for malaria infection necessitates drawing blood by fingerprick or venipuncture, which poses risks and limitations for repeated measurement. This study presents PCR detection of *Plasmodium falciparum *in human urine and saliva samples, and illustrates this potential application in genotyping malaria infections.

**Methods:**

Urine and saliva were obtained from 47 thick film positive and 4 negative individuals one day after collection of blood slides and filter paper blood spots. *P. falciparum *DNA was extracted from blood, urine and saliva, in separate groups, using the Chelex method or Qiagen DNEasy^® ^kit (urine and saliva only). Blood, urine and saliva extracts were subjected to PCR in separate batches. Amplicons from the various sample types were examined for MSP2 polymorphisms and restriction fragment patterns on DHFR amino acid codon 59.

**Results and discussion:**

Malaria infections exhibited primarily low-grade parasite densities, with a geometric mean of 775 asexual parasites/μl. Regularly matching polymorphic MSP2 genotypes were found between the corresponding urine, saliva and peripheral blood amplicons of each individual, with different inter-individual polymorphic genotypes. Amplicon yields were significantly dependent on DNA extraction method, parasite density and primer set (p < 0.001). A Qiagen^® ^kit extraction had more than 2× higher amplicon yield than the Chelex method, for both urine and saliva. Amplicon yields were 1.6 fold higher from saliva than urine. For each unit increase in log parasite density, the probability of amplicon enhanced 1.8 fold. Highest amplicon yields were obtained from the primer set with the shortest PCR product.

**Conclusion:**

*P. falciparum *infection is detectable by PCR on human urine and saliva samples. Subject to further refinement of extraction technique and amplicon yields, large-scale malaria parasite screening and epidemiological surveys could be possible without the need to collect blood and use of needles or sharps.

## Background

Endemic countries are adopting more effective strategies and treatment regimens for malaria control, through public-private sector initiatives such as the RBM [1]. As substantial impact is brought to bear on this formidably resilient disease [2,3], the increased importance of accurate diagnosis, epidemiological surveillance and research cannot be overemphasized. Use of efficient molecular tools is becoming widely integrated to afford more informative and comparable results in programmes such as national drug therapeutic efficacy monitoring [4,5]. Molecular genotyping by national drug therapeutic efficacy monitoring programmes to distinguish post-treatment recrudescence from new infections is becoming universal standard [6].

In accord with current knowledge of the parasite life cycle, detection or screening for malaria infection presupposes the drawing of blood by finger prick or venipuncture. The need to draw blood causes difficulties in certain communities with blood taboos and poses limitations for repeated measurement, especially in young children, who experience the highest malarial burden and constitute the mainstay sentinel group for most malaria epidemiological surveys. Inevitable use of needles or sharps poses a biohazard as well as extra workload and cost in field situations. The present study describes the use of urine and saliva samples for PCR detection of *Plasmodium falciparum *infection and illustrates potential application in genotyping malaria parasites.

## Methods

### Study area and population

The study was conducted in the vicinity of the Malaria Institute at Macha (MIAM), located 80 km north of Choma, in the southern province of Zambia. The resident population, which is estimated at 180,000, comprises subsistence farmers of the Batonga tribe. Willing subjects of all ages were included in the study.

### Study design and data collection

This prospective cross-sectional study explored the hypothesis that *P. falciparum *infection detectable by PCR on peripheral blood is also detectable by PCR on urine or saliva from the same human host. By prior appointment through local chiefs and headmen, communities assembled at central designated points of their choice, for malaria screening by microscopy. Thick films and filter paper (Whatman No 3 MM) blood blots were collected, with recording of axillary temperature and history of symptoms in the past 48 hours. Slides were examined at MIAM, followed by feedback to the community, including treatment of positives, the following day. On the day of feedback, whole urine and saliva samples (5 ml) were collected from all willing positive individuals, in sterile tubes, while some were blotted onto filter paper cuttings. The whole urine or saliva specimens were subsequently aliquoted into 1 ml replicate amounts in microcentrifuge tubes at the laboratory and either immediately extracted or stored at -20°C for later extractions. Additional blood samples were also collected on filter paper. Separate drying boards were used for urine, saliva and blood filter paper blots.

### *P. falciparum *DNA extraction

#### Chelex extractions

(a) Filter paper blots

Filter paper blots of saliva, urine and blood were extracted using the Chelex method [7,8].

(b) Whole urine and saliva

Whole urine or saliva (1 ml) was spun in a 1.5 ml microcentrifuge tube for three minutes at 14,000 rpm. The supernatant was aspirated out to the 0.1 ml mark and discarded. The pellet was then re-suspended in 1 ml of 1× PBS/1% saponin solution by gentle tapping and vortexing, and left at room temperature for 20 minutes. Following lysis, the sample material was spun for two minutes at 14,000 rpm and the supernatant aspirated and discarded. The pellet was re-suspended in 1 ml of 1× PBS, followed again by spinning for two minutes at 14,000 rpm. After aspirating and discarding of supernatant, a 100 μl suspension of 20% Chelex in autoclaved PCR-grade water was added and mixed thoroughly with the pellet. The mixture was then boiled for 13 minutes and finally spun for three minutes, at 14,000 rpm. The resultant supernatant, containing DNA, was carefully transferred into a pre-labelled 1.5 ml microcentrifuge tube, excluding Chelex, for immediate PCR, or stored at -20°C.

#### Commercial kit extraction (Qiagen DNeasy^® ^purification kit)

Whole urine or saliva sample (1 ml) was spun for 3 minutes at 14,000 rpm. After aspirating and discarding supernatant, DNA was extracted using the crude cell lysates protocol following the manufacturer's instructions. Kit Buffer ATL^® ^(200 μl) was used for the initial lysis step on the pellet and final DNA elution was carried out in 100 μl volumes.

### Genotyping of *Plasmodium falciparum *in urine and saliva

*P. falciparum *genotyping was performed on blood, urine and saliva extracts using nested PCR and MSP2 family-specific primers [9]. Nested PCR and allele-specific restriction enzyme digestion were employed for typing DHFR amino acid codon 59[10]. For comparison with standard DHFR primers, additional primer sets (U1/U2, U3/U4) were designed on the DHFR domain that define shorter primary (370 bp) and secondary (229 bp) amplicon (Table 1).

**Table 1:** Characteristics of primers used in nested PCR on *P. falciparum *DHFR. Lower case denotes nucleotide mismatch.

Reaction	Primer	Nucleotide location	Product size (bp)
Nest I	U1: 5'-GGAAATAAAGGAGTATTACCATG-3'	121..143	
	U2: 5'-TAAGGTTCTAGACAATATAACA-3'	393..372	273
Nest II	U3: 5'-GAAATGTAaTTCCCTAGATATGgAATATT-3'	144..172	
	U4: 5'-ATTTATCCTATTGCTTAAAGGT-3'	372..351	229
Nest I	M1: 5'-TTTATGATGGAACAAGTCTGC-3'	3..18	
	M5: 5'-AGTATATACATCGCTAACAGA-3'	645..625	643
Nest II	F: 5'-GAAATGTAATTCCCTAGATATGgAATATT-3'	144..172	
	M4: 5'-TTAATTTCCCAAGTAAAACTATTAGAgCTTC-3'	469..439	326

Primary and secondary PCR reactions for all loci were run in 25 μl volumes comprising 2 μl template (0.5 μl for laboratory standard clone genomic DNA), 0.25 μM primers (0.2 μM for MSP2), 1.5 mM magnesium chloride, 200 μM dNTP's, 1× PCR Buffer and 1U of Taq DNA polymerase. PCR annealing cycles for both primary and secondary MSP2 reactions comprised an initial two minute denaturation step at 94°C, followed by 25 cycles of denaturation at 94°C for 45 seconds, annealing at 61.1°C for 45 seconds and extension at 65°C for one minute. Final extension was at 65°C for two minutes. Annealing cycles for both both primary and secondary DHFR amplification consisted of initial denaturation at 94°C for two minutes, followed by 25 cycles of denaturation at 94°C for 45 seconds, annealing at 43.4°C for 45 seconds and extension at 65°C for one minute. The final extension step was at 65°C for two minutes. The PCR amplifications were performed in a Thermo Electron^® ^PX2 (HBPX2) thermal cycler. Polymorphisms and restriction fragment patterns were resolved by electrophoresis on 1.5% agarose gel (12 μl of sample per lane) and visualized by UV transillumination. Product for 3D7/IC and FC27 family-specific primers was run on separate gels to score amplicon yields. To facilitate between-sample comparison of MSP2 polymorphic patterns, the 3D7/IC and FC27 product was then loaded in duplex for each sample and run on a fresh set of gels.

#### Ethics

The study was approved by the national and Johns Hopkins University IRB as part of a baseline geographical and malariometric reconnaissance project at Macha. Patient participation was by informed consent of the patients themselves or guardians, in the case of children.

## Results

The 51 cases from whom assay samples derived consisted of 47 microscopy-positive and four microscopy negative individuals, aged from 1–54 years (median 11.0 years). Of the 47 that were positive by microscopy, malarial infections exhibited low-grade parasite density, with a median of 708 asexual parasites/μl (geometric mean [95% CI]: 775 [417–1439] asexual parasites/μl; range: 37 – 123026 asexual parasites/μl). All the infected individuals were asymptomatic, except for two febrile cases whose infections had the highest parasite density (mean axillary temperature, 36.85°C; median, 36.90°C; range: 35.60°C – 41.00°C).

### MSP2 genotyping of *P. falciparum *on urine and saliva samples

**Figure 1:**
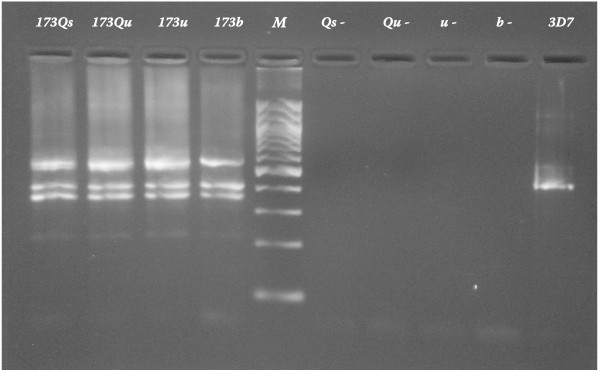
Showing *P. falciparum *MSP2 amplicon from saliva (173Qs), urine (173Qu, 173u) and blood (173b) samples of patient 173. Qs and Qu denote saliva and urine samples extracted by Qiagen^® ^commercial kit (crude cell lysate protocol), while u denotes whole urine sample extracted by the Chelex method. Qs-, Qu-, u- and b-, denote amplicon from corresponding extracts of saliva, urine and blood donated by thick film negative healthy control. 3D7, amplicon from positive control laboratory standard; M, 100 bp DNA ladder. Extraction of urine replicate sample 173u was performed on 20.02.06, while Qiagen extractions were carried out on 09.02.06. Identical MSP2 alleles are apparent in amplicon from urine, saliva and blood samples of patient 173, as compared to 3D7 lab standard.

**Figure 2:**
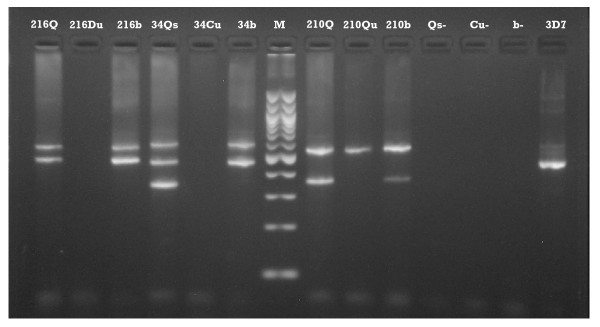
Amplicon from saliva, urine and blood extracts of field samples 216, 34 and 210. Matching MSP2 alleles are seen in saliva (Qs) and blood (b) amplicon from each individual. In contrast, between-patient polymorphic differences are evident, reflecting diverse infections. Urine samples from these patients did not amplify, except that of 210 (210Qu). Qs, Qu denote Qiagen saliva and urine extracts, respectively, by crude lysate approach; Cu denotes Qiagen urine extracts, by cultured animal cells protocol; Du denotes Chelex direct extraction on whole urine. Qs-, Cu-, b- were corresponding extracts of saliva, urine and blood samples from healthy negative control, while 3D7 was positive control laboratory standard.

Saliva, urine and blood extracts were subjected to nested PCR in separate batches using MSP2 family-specific primers and then loaded in adjacent lanes for each patient during electrophoresis. Amplicons from saliva and urine extracts exhibited MSP2 polymorphic patterns as well as multiplicity of infection that was regularly identical to blood sample extracts from the same individual (Figure 1 and 2). As expected MSP2 amplicons from different patients were polymorphic (Figure 2).

### *P. falciparum *DHFR genotyping on urine and saliva samples

**Figure 3:**
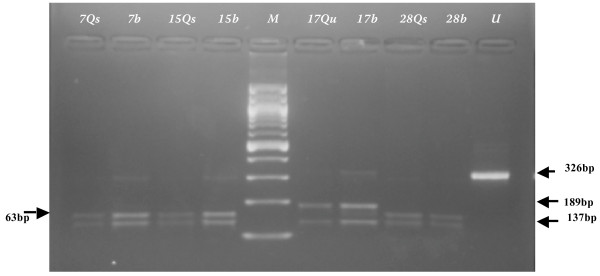
*Xmn *I digest of DHFR F-M4 amplicon from saliva, urine and blood samples of patients 7, 15, 17 and 28. Qs, Qu denotes saliva and urine samples extracted by Qiagen^® ^kit (crude cell lysate protocol). Blood samples were extracted on different days by Chelex from dry filter paper blots. U is undigested DHFR F-M4 amplicon. Identical restriction fragment patterns are evident on different extracts from each patient. Patients 7, 15 and 28 were infected with parasites carrying the mutated (antifolate resistance associated) allele (coding for DHFR Arg-59). Patient 17 carried mixed wild type (Cys-59) and mutated (Arg-59) variants, of which the mutated form was barely discernible in the urine amplicon. Incomplete digestion is present in some amplications, with a band at 326 bp.

As with MSP2 polymorphisms, restriction fragment results for the DHFR amplicon flanking amino acid codon 59 showed that the infection detected in urine/saliva PCR was the same as that found in the corresponding blood amplicon for each individual, whereas between-patient differences were apparent (Figure 3). The genotyping results showed more infections with the mutated Arg-59 allele (64.7%) including 17.6% mixed mutated and wild variants in the same individual.

### Amplicon yield

Amplicon yield differed considerably by DNA extraction method (Additional File 1). Some of the 47 thick-film positive samples were PCR-negative, with different percent yields amongst primer sets (Additional File 1). Amongst the four thick film negative field samples two had positive PCR amplifications.

Aliquots of whole saliva and urine extracted by the Qiagen commercial kit afforded 2.6× (odds ratio [95% CI]: 2.609 [1.269–5.331], p = 0.008) and 2.3× (odds ratio [95% CI]: 2.289 [1.281–4.091], p = 0.005) better amplification success, respectively, than the Chelex approach on corresponding specimens. Saliva extracts generally gave 1.6 fold better amplicon yield than corresponding urine extracts (odds ratio [95% CI]: 1.559 [1.046–2.324], p = 0.029).

In addition to extraction method, amplicon yield was also significantly dependent on parasite density and primer set. For each unit increase in log parasite density, the probability of amplification increased 1.8 fold (odds ratio [95% CI]: 1.826 [1.529–2.181], p < 0.001). The DHFR primer set U1-4 afforded the best amplification success, while lowest amplicon yields were obtained with FC27, which was 18.5× less likely to amplify than U1-4. The FC27 primary amplicon is around 750 bp in length in contrast to 370 bp for U1-4. Using primers U1-4, the probability of amplification was not significantly different between commercial (Qiagen^®^) kit saliva extracts and standard chelex-extracted filter paper blood (odds ratio [95%CI]: 0.522 [0.133–2.049], p = 0.351). For kit-extracted urine, there was a barely significant difference (odds ratio [95% CI]: 0.258 [0.068–0.982], p = 0.047). However, chelex-extracted saliva and urine were still 9.6× (odds ratio [95% CI]: 0.104 [0.017–0.650], p = 0.015) and 10.2× (odds ratio [95%CI]: 098 [0.026–0.366], p = 0.001) less likely to amplify, respectively, than corresponding blood extracts.

## Discussion

Current malaria detection and epidemiological surveys depend on drawing blood, which entails risks and often poses difficulties in young children and certain communities with blood taboos. Because they are the most vulnerable to infection and disease, young children tend to constitute the *de facto *sentinel group used for most malaria surveys. The present study shows for the first time that *P. falciparum *infection can be detected by PCR on saliva and urine samples from the human host. Nested PCR with *P. falciparum *MSP2 family-specific primers illustrates that the infection detected in saliva or urine samples is regularly identical to that found in the corresponding peripheral blood of the same individual. In contrast, between-patient MSP2 polymorphic variations were evident among the study samples.

These findings open up possibilities to obviate the drawing of blood and the use of needles or sharps during large scale screening or epidemiological surveys for malaria infection. Potential advantages are also envisaged for cohort or follow-up studies entailing repeated sampling or testing for *P. falciparum *infection. Molecular genotyping in clinical trials and drug or vaccine efficacy monitoring programmes could be significantly facilitated by such a bloodless approach. The fact that amplification was achieved on infections of low-grade parasite density appears to bode well for potential application of this principle.

There is an important need to refine DNA isolation technique and maximize amplicon yields to attain levels possible with blood samples. It was clear that apart from parasite density and extraction method, the locus or primer set used significantly influenced the probability of PCR amplification from a given infection. This presumably reflects differences in primer annealing properties and PCR conditions. However, it was notable, firstly, that the PfDHFR primer set U1-4, which flanks the shortest amplicon fragment, achieved the highest amplification success. Secondly, the U1-4 amplicon yields were actually similar between Qiagen^® ^saliva extracts and blood, and only marginally different for Qiagen^®^-extracted urine. This would seem to suggest that DNA may be degraded in saliva or urine samples such that chances of amplification are greater with short PCR fragments. Comparisons with larger sample sizes would be needed to clearly establish the relative influence of product length on amplicon outcome.

Identifying the source of malarial DNA in human urine or saliva could also aid in development of protocols for maximizing amplicon yields and assay detection sensitivity. At present, the DNA source is still unclear and not directly inferrable from the current study. Speculations range from free molecular complexes released by lysed cells, to parasites in trace amounts of blood escaping into urine and saliva. Comparisons of experimental DNA extraction protocols in this study would seem to suggest an intracellular origin for the DNA, either in erythrocyes or trapped in macrophages. Future work will attempt to examine this aspect.

## Conclusion

This study illustrates for the first time, the principle of detecting *P. falciparum *infection by PCR on human urine and saliva samples. Subject to refinement of extraction technique and amplicon yields, future large scale malaria screening or epidemiological surveys could be possible without the need to draw blood or the use of needles or sharps.

## Authors' contributions

SM was the principal investigator responsible for the study design, data collection, analysis and manuscript preparation, and initiated the idea of testing human saliva. CS carried out field data collection and microscopy for the study and assisted with laboratory assays and management. PET provided invaluable technical guidance and critical input to the protocol and manuscript. CJS contibuted important technical advice and contibuted to the protocol and manuscript. DJS was the overall project leader who founded the idea of testing human urine as well as input vital technical guidance in the development of the proposal, data collection, analysis and manuscript preparation.

## Supplementary Material

Additional File 1 PCR amplicon yield by DNA extract type and primer set Click here for file
