# Supplementary material for: PCR detection of Plasmodium falciparum in human urine and saliva samples
Source: Malar J. 2006 Nov 8;5:103. doi: 10.1186/1475-2875-5-103 (PMC1654175; doi:10.1186/1475-2875-5-103)
Supplement: Additional File 1 — PCR amplicon yield by DNA extract type and primer set [file 1475-2875-5-103-S1.doc]

Additional File 1

PCR amplicon yield by DNA extract type and primer set (Qs, saliva Qiagen kit extract ; s, saliva chelex extract; Qu, urine Qiagen kit extract ; u, urine chelex extract ; b, blood chelex extract).

| Patient # | Parasite density/µl | Extract | PCR amplicon | | | |
| --- | --- | --- | --- | --- | --- | --- |
|  |  |  | MSP2 | | DHFR | |
|  |  |  | 3D7/IC | FC27 | FM4 | U3U4 |
| 1 | 3490 | s | Yes | Yes | Yes | Yes |
|  |  | b | Yes | Yes | Yes | Yes |
| 2 | 0 | Qs | No | No | No | No |
|  |  | s | No | No | No | No |
|  |  | Qu | No | No | No | No |
|  |  | u | No | No | No | No |
|  |  | b | No | No | No | No |
| 4 | 37 | Qs | No | No | No | Yes |
|  |  | u | No | No | No | No |
|  |  | b | Yes | Yes | Yes | Yes |
| 6 | 464 | s | No | No | Yes | No |
|  |  | u | No | No | No | Yes |
|  |  | b | Yes | Yes | Yes | Yes |
| 7 | 1273 | Qs | No | No | Yes | Yes |
|  |  | Qu | No | No | No | Yes |
|  |  | b | Yes | No | Yes | Yes |
| 11 | 546 | Qs | No | No | Yes | Yes |
|  |  | Qu | No | No | Yes | Yes |
|  |  | u | No | No | No | No |
|  |  | b | Yes | Yes | Yes | Yes |
| 13 | 110 | Qs | No | No | No | Yes |
|  |  | Qu | No | No | No | Yes |
|  |  | b | Yes | Yes | Yes | Yes |
| 15 | 13308 | Qs | No | No | Yes | Yes |
|  |  | Qu | No | No | No | Yes |
|  |  | u | No | No | - | - |
|  |  | b | Yes | Yes | Yes | Yes |
| 16 | 39 | Qs | No | No | No | No |
|  |  | Qu | Yes | Yes | No | Yes |
|  |  | u | No | No | - | - |
|  |  | b | Yes | Yes | Yes | Yes |
| 17 | 2549 | Qs | No | No | No | Yes |
|  |  | Qu | No | No | Yes | Yes |
|  |  | u | Yes | Yes | - | - |
|  |  | b | Yes | Yes | Yes | Yes |
| 21 | 74 | Qs | No | No | No | Yes |
|  |  | Qu | No | No | No | Yes |
|  |  | b | Yes | No | Yes | Yes |
| 22 | 928 | Qs | No | No | No | Yes |
|  |  | u | No | No | No | Yes |
|  |  | b | Yes | No | Yes | Yes |
| 23 | 21687 | Qs | No | No | No | Yes |
|  |  | Qu | No | No | No | Yes |
|  |  | b | Yes | Yes | Yes | Yes |
| 24 | 40 | Qs | No | No | No | No |
|  |  | Qu | No | No | No | Yes |
|  |  | b | No | No | Yes | Yes |
| 27 | 306.2 | Qu | No | No | No | No |
|  |  | u | No | No | No | No |
|  |  | b | No | No | No | No |
| 28 | 32320 | Qs | Yes | Yes | Yes | Yes |
|  |  | Qu | No | No | No | No |
|  |  | u | No | No | - | - |
|  |  | b | Yes | Yes | Yes | Yes |
| 29 | 39 | Qs | No | No | No | Yes |
|  |  | Qu | No | No | No | Yes |
|  |  | b | No | No | No | Yes |
| 32 | 73 | Qs | Yes | No | Yes | Yes |
|  |  | s | No | No | No | - |
|  |  | Qu | No | Yes | No | Yes |
|  |  | u | No | No | No | - |
|  |  | b | Yes | Yes | Yes | Yes |
| 34 | 451 | Qs | Yes | Yes | Yes | Yes |
|  |  | Qu | No | No | No | Yes |
|  |  | u | No | Yes | - | - |
|  |  | b | Yes | Yes | Yes | Yes |
| 35 | 639 | Qs | No | No | No | Yes |
|  |  | u | No | No | No | No |
|  |  | b | Yes | Yes | Yes | Yes |
| 38 | 815 | Qs | No | No | Yes | Yes |
|  |  | Qu | No | No | Yes | Yes |
|  |  | b | Yes | No | Yes | Yes |
| 41 | 0 | b | No | No | No | No |
| 45 | 5920 | Qs | Yes | Yes | Yes | Yes |
|  |  | Qu | No | No | No | Yes |
|  |  | u | No | No | - | - |
|  |  | b | Yes | No | Yes | Yes |
| 50 | 124272 | Qu | Yes | Yes | Yes | Yes |
|  |  | u | Yes | Yes | No | Yes |
|  |  | b | Yes | Yes | Yes | Yes |
| 51 | 0 | Qs | Yes | Yes | Yes | Yes |
|  |  | s | No | No | No | - |
|  |  | Qu | No | No | No | Yes |
|  |  | u | No | No | No | Yes |
|  |  | b | Yes | Yes | No | Yes |
| 52 | 77 | Qu | No | No | No | No |
|  |  | u | No | No | No | No |
|  |  | b | No | No | No | No |
| 66 | 192 | Qs | No | No | No | Yes |
|  |  | Qu | Yes | Yes | Yes | Yes |
|  |  | b | Yes | Yes | Yes | Yes |
| 69 | 937 | Qs | No | No | No | No |
|  |  | u | No | No | No | Yes |
|  |  | b | Yes | No | Yes | Yes |
| 83 | 64 | S | No | No | No | No |
|  |  | b | No | No | Yes | Yes |
| 84 | 574 | Qs | No | No | No | Yes |
|  |  | u | No | No | No | Yes |
|  |  | b | No | No | Yes | Yes |
| 86 | 2459 | Qs | No | No | No | No |
|  |  | u | No | No | No | Yes |
|  |  | b | Yes | Yes | Yes | Yes |
| 90 | 784 | Qs | No | No | No | Yes |
|  |  | u | No | No | No | Yes |
|  |  | b | No | No | Yes | Yes |
| 101 | 0 | b | No | No | No | No |
| 108 | 815 | s | No | No | No | Yes |
|  |  | u | Yes | Yes | Yes | Yes |
|  |  | b | Yes | Yes | Yes | Yes |
| 111 | 37231 | S | No | No | Yes | No |
|  |  | b | Yes | Yes | Yes | Yes |
| 130 | 155 | Qs | No | No | No | Yes |
|  |  | u | No | No | No | No |
|  |  | b | Yes | No | Yes | Yes |
| 158 | 2717 | Qs | No | No | No | Yes |
|  |  | u | No | No | No | No |
|  |  | b | Yes | Yes | Yes | Yes |
| 173 | 22890 | Qs | Yes | Yes | Yes | Yes |
|  |  | Qu | Yes | Yes | Yes | Yes |
|  |  | u | Yes | Yes | Yes | Yes |
|  |  | b | Yes | Yes | Yes | Yes |
| 189 | 30731 | Qs | No | No | Yes | Yes |
|  |  | s | No | No | Yes | Yes |
|  |  | u | No | No | Yes | Yes |
|  |  | b | No | No | Yes | Yes |
| 210 | 1015 | Qs | Yes | Yes | Yes | Yes |
|  |  | s | No | No | No | - |
|  |  | Qu | No | No | No | No |
|  |  | u | No | No | Yes | - |
|  |  | b | Yes | Yes | Yes | Yes |
| 216 | 13463 | Qs | Yes | Yes | Yes | Yes |
|  |  | s | No | No | No | No |
|  |  | u | No | No | - | - |
|  |  | b | Yes | Yes | Yes | Yes |
| 246 | 881 | Qs | No | No | No | Yes |
|  |  | s | Yes | Yes | No | Yes |
|  |  | Qu | - | - | - | Yes |
|  |  | u | No | No | No | Yes |
|  |  | b | Yes | Yes | Yes | Yes |
| 404 | 111 | s | - | - | - | No |
|  |  | u | No | No | No | No |
|  |  | b | Yes | No | Yes | Yes |
| 411 | 1970 | Qs | Yes | Yes | Yes | Yes |
|  |  | s | No | No | No | No |
|  |  | Qu | No | No | No | No |
|  |  | u | No | No | No | - |
|  |  | b | Yes | Yes | Yes | Yes |
| 413 | 38 | s | No | No | No | - |
|  |  | Qu | Yes | Yes | Yes | - |
|  |  | u | No | No | No | - |
|  |  | b | Yes | Yes | Yes | - |
| 443 | 159 | Qs | No | No | No | Yes |
|  |  | u | No | No | No | No |
|  |  | b | Yes | Yes | Yes | Yes |
| 450 | 262 | Qs | Yes | No | Yes | Yes |
|  |  | u | No | No | No | Yes |
|  |  | b | Yes | No | Yes | Yes |
| 453 | 152 | Qs | No | No | No | Yes |
|  |  | u | No | No | No | No |
|  |  | b | No | No | No | Yes |
| 482 | 117 | Qs | No | No | No | Yes |
|  |  | u | No | No | No | No |
|  |  | b | Yes | Yes | Yes | Yes |
| 486 | 466 | Qs | No | No | No | Yes |
|  |  | u | No | No | No | Yes |
|  |  | b | Yes | Yes | Yes | Yes |
| 534 | 1385 | Qs | No | No | No | Yes |
|  |  | Qu | No | No | Yes | Yes |
|  |  | b | Yes | Yes | Yes | Yes |
